# Translation and validation of the German version of the Bournemouth questionnaire for low back pain

**DOI:** 10.1186/2045-709X-21-32

**Published:** 2013-09-26

**Authors:** Celina Blum-Fowler, Cynthia Peterson, Johanna Forrer McChurch, Yann Le Clech, B Kim Humphreys

**Affiliations:** 1 Chiropractic Medicine Department, Faculty of Medicine, University of Zürich and Orthopaedic University Hospital Balgrist, Forchstrasse 340, 8008 Zürich, Switzerland; 2 Swiss Academy for Chiropractic, Sulgenauweg 37 3008 Bern, Switzerland

**Keywords:** Bournemouth questionnaire, Outcome assessment, Low back pain, Chiropractic, Validity of results

## Abstract

**Background:**

Finding the best outcome measures for research and quality assurance purposes in terms of validity, sensitivity to change, length and ease of completion is crucial. The Bournemouth questionnaire for neck pain patients was recently translated and validated into German and found to be more sensitive to change than other commonly used questionnaires. However, the low back pain version is not yet available in German. Therefore the purpose of this study was to translate and validate the Bournemouth Questionnaire (BQ) for low back pain (LBP) into German.

**Methods:**

The translation was done in 4 steps, translated and back-translated by two independent people and adapted and approved by an expert committee. Face validity was then done by 30 people who checked the questionnaire for comprehension. Test-retest reliability (reproducibility) was tested using 30 stable back pain patients. Internal consistency was tested using 108 low back patients. External construct validity, external longitudinal validity and responsiveness was tested against the German versions of the Oswestry Disability Index (ODI) and the SF-36 questionnaire using 108 patients from 5 different chiropractic clinics.

**Results:**

The BQ showed high test-retest reliability (ICC > 0.91) for all items and strong internal consistency (Cronbachs alpha = 0.86 at baseline and 0.94 at 4 weeks).

The BQ demonstrated good external construct and longitudinal construct validity with established measures. The effect sizes of the BQ were high and comparable with established measures.

External construct validity and external longitudinal construct validity showed significant correlation for all 7 scales of the BQ with the relevant scales of the other questionnaires with one exception. External responsiveness results showed higher effect sizes for the BQ items and total score indicating better sensitivity to change than the compared measures.

**Conclusion:**

The BQ for LBP was successfully translated and adapted into German. It was successfully tested for validity, consistency, and responsiveness against the German versions of the ODI and the SF-36. It is shorter, covers more domains than the ODI and is more sensitive to change than the other questionnaires.

## Background

Back pain is a very common complaint with as many as 75-85% of all people, at some stage in their life, being affected [1,2]. In the last twenty years there has been an alarming increase in back pain disability, even described as reaching epidemic proportions [3]. In Switzerland, low back pain is the most prevalent health problem with a recent study showing that 47% of women and 39% of men suffered from various back problems in the preceding 4 weeks [4]. Furthermore, medical direct costs due to LBP corresponded to 6.1% of the total healthcare expenditure in Switzerland in 2005 [4]. This shows the need for and importance of research into the outcomes from the various treatments for low back pain in order to identify best practice.

Evidence-based practice should include the regular use of outcome measures to monitor the progress of individual patients and the results of the practice or practitioner as a whole. Outcome measurement questionnaires are commonly subjective functional behavioural measures, filled out by the patient him/herself. An essential component of any outcome measure (including questionnaires) is the reliability and validity of the instrument [2] and its sensitivity to detect clinically significant change in the condition [5,6]. When it comes to measuring back pain, pain itself is of course the most important symptom. However, pain is a multidimensional, individual experience or behaviour with a number of sensory affective, cognitive, behavioural and social aspects [7]. Therefore, it is not enough to simply measure pain levels.

Because functional status is a very important outcome for back pain patients, there are already good self-report outcome measures testing functional status as in everyday living, household and work tasks and leisure activities [8,9]. There are also numerous instruments available to test the psychosocial profile of back pain patients, as in psychological influences, social roles, well-being and overall improvement [5]. Thus it is possible to measure pain with all of its various aspects using the outcome measures available, but selecting the right outcome measures to be able to cover all the different dimensional aspects of the pain experience is often impractical while choosing shorter and simpler measures may not reflect the true complexity of the complaint. There are also multidimensional health status Instruments available, but they lack the condition specificity of low back pain [10,11].

These observations led Bolton and Breen in 1999 to develop the Bournemouth Questionnaire (BQ) for low back pain, a comprehensive and short form multidimensional back pain measure suitable for use in both the clinical setting and in clinical trials [5]. The questionnaire contains 7 items which cover all of the important dimensions of pain and functioning. Each item or domain contains a question and a scale from 0–10. The questions relate to pain, physical disability, social disability, anxiety, depression, fear avoidance thoughts in relation to work and the own ability to control pain. The BQ has been tested and proved to be reliable, valid and responsive to change [5]. It is not only short and multidimensional but also easy to fill out by patients and easy to evaluate by clinicians. These characteristics make the BQ a favourable instrument for use in the clinical setting as well as research studies.

The BQ was subsequently modified for patients suffering from neck pain [12] and this neck pain version was recently translated and validated into German [13]. However, the BQ for low back pain patients has not been available in the German language. Therefore, the purpose of this study was to translate and validate the BQ for low back pain patients into German based on documented translation and cross cultural adaptation processes for self-report measures [14,15].

## Methods

Ethics approval for this study was obtained by the Canton of Zürich ethics review board (KEK-ZH-Nr.2010-0252/5). This study was approved under the same ethics proposal as for the ‘Translation and Validation of the German version of the Bournemouth Questionnaire for Neck Pain’ published in 2012 [13] as the methodology is extremely similar. However, totally different patient populations were used for each study. This current study only used patients presenting with low back pain whereas the previous study only used patients presenting with neck pain. Patients for the two studies were also recruited from different practice locations in Switzerland.

**Figure 1:**
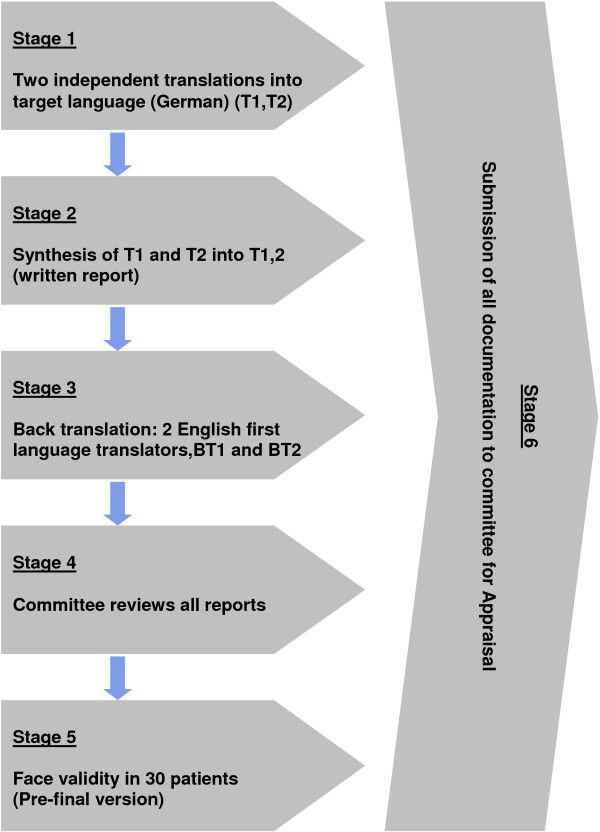
Translation and cross-culture adaptation sequence.

The translation and cross cultural adaptation process of the BQ LBP was based on the guidelines of Beaton, Bombardier et al. [14]. The six different stages that were needed for the process are seen in Figure 1.

### Stage 1 (forward translation)

For the forward translation from English into German, two independent native German speaking translators were used to translate the BQ (LBP) into the target language, German (T1 and T2). One of the translators was a clinician and therefore aware of the concepts that are being measured with the BQ (LBP) and the other translator was a language specialist with no chiropractic or medical background.

### Stage 2 (Synthesis of T1 and T2 into T1,2)

The two translators had to then agree on one new consensus version of the translation (T1,2). This consensus version was overseen by the expert committee overseeing the project.

### Stage 3 (back translation)

For the back translation from German into English, two English first language translators (BT1 and BT2) were required. They both grew up in English speaking countries with bilingual parents and have now been living in Switzerland for over 20 years, thus being totally bilingual. They both independently translated T1,2 back into English. They were blinded to the original English version of the BQ during this process.

### Stage 4 (Expert committee)

The committee consisted of methodologists, health professionals, translators and a language professional. The committee reviewed all the translations (T1 and T2, T1,2, B1 and B2) and the written report comparing the back-translations with the forward-translation T1,2. Based on those translations they developed the pre-final version.

### Stage 5 (Face validity)

The pre-final version of the questionnaire was tested on 30 people. Each completed the questionnaire and was then asked the meaning of each questionnaire item as well as whether or not they had problems with the questionnaire format, layout, instructions or response scales. Any difficulties were noted and include in the final report. A detailed report written by the interviewing person, including proposed changes of the pre-final version based on the results of the face validity test was then submitted to the expert committee.

### Stage 6 (committee appraisal)

The final version of the German BQ LBP was developed by the committee based on the results of the face validity testing and the written report. Thus all stages 1–6 were successfully completed. The final version of the BQ for LBP patients can be found in Additional file 1.

### Test-retest reliability

The BQ LBP questionnaire was tested for reliability using two German BQ LBP versions administered to 31 students with low back pain at the Eidgenösische Technische Hochschule (ETH) Zurich. It was essential that no change or treatment occurred in between the two administrations. Therefore a lecture was used for the testing with the two versions given before and after the 2 hour class.

The 7 questionnaire item domains were given in different random order for the second administration to avoid the students memorizing their initial responses [5].

### Validation

The purpose of cross-cultural adaptation is to try and ensure consistency in the content and face validity between the original and the translated versions of a questionnaire. However, it does not ensure that the questionnaire has construct validity. Content validity of the BQ LBP questionnaire was previously evaluated on the original English version, and was therefore not tested in this study. Additional testing was done to evaluate construct validity, however [16,17]. This additional testing of the instrument should be done in the same population where it would be used [12]. The BQ is commonly used as an outcome measure for neck and low back pain patients being treated by chiropractors in the UK, where it was developed, and is also used in other countries [12,16]. Thus 108 low back pain patients from five different chiropractic practices were asked to fill in the new German version of the BQN, the German version of the Oswestry Disability Index (ODI) [9] and the German version of the SF-36 Health Survey [10] prior to the start of their chiropractic treatment. Four weeks later each patient had to complete the 3 questionnaires again. The questionnaires were given to them in the practice or sent by post with an addressed and stamped return envelope. Those patients who received them in the practice filled them in immediately. Those who received them by post were allowed one week to return them. If the questionnaires were not returned within 1 week, the patients were called by phone and reminded to return the completed questionnaires. If necessary, the questionnaires were resent to the patients. The ODI and SF-36 were selected for comparison to the BQ LBP questionnaire as they contain similar subscales. To compare these three questionnaires, each one was broken down into its component subscales. Table 1 shows the matching of the various subscales on the ODI and SF-36 questionnaires with the seven subscales on the Bournemouth low back pain questionnaire.

**Table 1:** Matching of the subscales for the BQ, Oswestry and SF-36 questionnaires

**BQ subscale**	**Oswestry subscale**	**SF-36 subscale**
Pain	Pain (quest 1)	Pain: Section 7 (quest 21)
Physical function	Phys. Funct. (quests 2-7)	Phys. Funct. Section 3 (quests 3-12)
Social activity	Social Activity (quests 8-10)	Social Funct. (quest 20)
Anxiety		Emotional well-being: Section 9 (quests 23-31)
Depression		Energy/Fatigue: Section 5 (quests 17-18)
Work-related fear avoidance		Work: Section 4 (quests 13-16)
Pain locus of control		General Health: Section 1 (quest 1)

### Statistical analysis

Test-retest reliability of the BQN was evaluated using the two way mixed Intra-class Correlation Coefficient (ICC) [10,13,16]. Values of ≥ 0.5 are considered good [18]. The internal consistency of the BQN, which measures the degree to which items that make up the total score are all measuring the same underlying attribute, was assessed using Cronbach α [10,13,16]. A value ≥ 0.7 is acceptable but values ≥ 0.8 are preferred [18].

External construct validity shows the extent to which the BQN’s scores concord with the scores of other instruments measuring the same theoretical hypotheses of the concepts under consideration [13]. This was done using the Pearson’s correlation coefficient comparing the 7 scales and total score of the BQN with the ODI as well as the BQN with the SF-36 for answers given at baseline (pre-treatment) and at 4 weeks after the start of treatment [14]. External longitudinal construct validity was determined with Pearson’s correlation of the change scores of the various scales comparing the BQN with the other two questionnaires over the 4 week treatment period.

The sensitivity to change over time of the three questionnaires was assessed with the standardized response mean (SRM). The average change in scores for each scale was divided by the standard deviation of the score changes [13,17].

## Results

For the 31 students who participated in the test-retest reliability part of the study, their mean age was 22.7 (SD = 3.8) and 66.7% were female. For the 108 chiropractic patients with baseline and 4 week outcome data their mean age was 45.91 (SD = 16.09) and 62.5% were female. There was no significant age difference between the genders. The mean total baseline score for the BQ LBP was 28.36 (SD = 13.85) or 40.51% of the maximum score. For the Oswestry questionnaire, the mean baseline total score was 11.06 (SD = 7.33) or 22.12% of the maximum score. The mean baseline score for the SF-36 was 61.12 (SD = 16.60) or 61.12% of the maximum possible score.

### Test-retest reliability of the German BQ LBP

Table 2 shows the results of the test-retest reliability. The ICC values were above .91 and highly significant for all 7 domains of the BQ LBP indicating acceptable agreement for all scales and the total score.

**Table 2:** Test-retest reliability for the German Bournemouth questionnaire for low back pain patients

**Question**	**ICC**	**95% CI**	**P =**
1	.96	.92 to .98	.0001
2	.91	.81 to .96	.0001
3	.92	.83 to .96	.0001
4	.93	.85 to .97	.0001
5	.97	.94 to .99	.0001
6	.96	.92 to .98	.0001
7	.96	.92 to .98	.0001
Total Score	.99	.97 to .99	.0001

31 patients tested.

### Internal consistency of the German BQ LBP

The item-corrected total correlations for the German version of the BQ LBP questionnaire are shown in Table 3. All values are well above the cut-off point of 0.3 [18] which means that all of the seven scales (domains) contribute to the overall score. The Cronbach α was .86 at baseline for the total pre-treatment scores and .94 for the total post-treatment scores indicating acceptable consistency.

**Table 3:** Internal consistency of the German version of the BQ LBP questionnaire

	**Item-corrected total correlations pearson’s *r***							
**Domain (Item)**	**1**	**2**	**3**	**4**	**5**	**6**	**7**	**Cronbach’s alpha: Total Score**
Pre-treatment	.65	.72	.71	.60	.53	.62	.61	.86
Post-treatment	.81	.85	.76	.81	.75	.82	.75	.94

(*BQ* Bournemouth questionnaire, *LBP* low back pain).

### External construct validity and external longitudinal construct validity

The results for the external construct validity comparing the 7 domains on the BQ LBP with the similar domains on the Oswestry and SF-36 questionnaires (Table 1) both at baseline and at 4 weeks are shown in Table 4. All correlations, with one exception, were statistically significant at p < 0.02. The pain locus of control at baseline (domain 7 of the BQ) did not have a significant positive correlation with the similar domain on the SF-36 at baseline. The Oswestry questionnaire does not contain this particular domain so no comparison could be made. Table 5 shows the results for the external longitudinal construct validity comparing the German BQ LBP questionnaire with the Oswestry and SF-36 questionnaires. Statistically significant positive correlations were found for all 7 BQ LBP domains.

**Table 4:** External construct validity of items on the German BQ LBP

**BQ scale**	**Oswestry pre- treatment (*r)***	**Oswestry post-treatment *(r)***	**SF-36 pre-treatment *(r)***	**SF-36 post-treatment *(r)***
Pain	.51	.79	.55	.57
Physical function	.51	.78	.54	.55
Social function	.50	.72	.30	.54
Anxiety	.55		.53	.68
Depression	.52		.56	.44
Work-related fear avoidance	.66		.24	.24
Pain control	.24		.17*	.29
Total score	.59	.82	.67	.77

* = not statistically significant. All other correlations are significant at p < 0.02.

**Table 5:** External longitudinal construct validity of the German BQ LBP compared with the Oswestry and SF-36 questionnaires

**BQ neck scale**	**Oswestry Pearson *r *(significance)**	**SF-36 Pearson *r *(significance)**
Pain	.56 (.001)	.34 (.0001)
Physical function	.52 (.001)	.46 (.0001)
Social function	.45 (.001)	.24 (.012)
Anxiety		.44 (.0001)
Depression		.31 (.001)
Work-related fear avoidance		.25 (.01)
Pain control		.19 (.049)

### Standardized Response Mean (SRM)

As can be seen in Table 6, the BQ LBP questionnaire demonstrated greater responsiveness compared to the Oswestry and SF-36 for all 7 domains.

**Table 6:** Standardized response means for the German BQ LBP compared to the Oswestry and SF-36 questionnaires

**Scale**	**BQN**	**Oswestry**	**SF-36**
Pain	.79	.52	.67
Physical function	.66	.59	.42
Social function	.56	.45	.18
Anxiety	.64		.40
Depression	.41		.21
Work-related fear avoidance	.69		.44
Pain control	.54		.21

## Discussion

The translation and cultural adaptation of the German version of the Bournemouth Questionnaire for low back pain patients, although a long and tedious multi-step process, was done successfully according to published guidelines [14,15]. Testing of the German version of the BQ LBP questionnaire shows that it is reliable, valid and more sensitive to change over time compared to the ODI and SF-36. Both of these commonly used questionnaires contain similar domains and have also been translated and validated into German. These results are identical to those found in the translation and validation of the BQ into German for neck pain patients and into Danish for low back pain patients [13,16]. Advantages of the BQ LBP questionnaire over the Oswestry and SF-36 questionnaires are as follows: 1) it contains the 7 important domains included in the biopsychosocial model of back pain, whereas the Oswestry only contains three of these domains (Table 1) [12]; 2) it is much shorter than the SF-36 and slightly shorter than the Oswestry questionnaires; 3) it is more sensitive to change on all domains compared to the other two questionnaires, 4) it is very easy to score. It can now be used in routine clinical practice to monitor patient outcomes or for research purposes in German speaking countries.

The SF-36 questionnaire was selected for comparison with the BQ LBP questionnaire because it is a multidimensional, commonly used questionnaire for low back pain patients and has been translated and validated into German [10]. However, scoring the SF-36 was particularly challenging when using it in this study for comparison with the BQ LBP and Oswestry questionnaires. The recommended scoring system for the SF-36 is that each of the 8 domains has a range of scores from 0–100. A higher score on the SF-36 indicates better health, whereas a higher score on the BQ LBP and Oswestry questionnaires indicates worse health. Thus a score of 100 would be the best possible function on that domain in the SF-36. In order to avoid negative correlations and confusing results, the SF-36 scoring system was reversed for this study so that a higher score was a worse outcome, the same as for the BQ and Oswestry questionnaires. The translation and validation of this same questionnaire into Danish also used the SF-36. Although the issue of how they scored the SF-36 is not mentioned in their methods section, the results only show good positive correlations [16]. Thus a modification of the usual scoring system along these same lines must have occurred.

Although the baseline total score for the SF-36 after reversing the usual scoring system was over 61% of the maximum score, providing plenty of room to show improvement over time, it was less sensitive to change in condition after 4 weeks for all domains compared to the BQ LBP which had a total baseline score of just over 40% of the maximum possible score. The Oswestry questionnaire had the lowest baseline total score of only 22% of the maximum possible score, making it susceptible to the so-called ‘floor effect’. It did not leave much room to show improvement. The Oswestry questionnaire is often used for very acute patients and therefore may not have been applicable to some of the chiropractic patients. The Oswestry questionnaire asks patients about their condition at the moment rather than on average. The BQ asks the patient to rate their pain, disability etc.’ on average over the past week’. However, this too can be problematic for the very acute patient who has not suffered with his/her low back condition for this length of time. Some of the study participants commented on the difficulty in answering the BQ questions when their symptoms were of a very short duration. However, this was only a problem for the baseline measurements. This situation may be more common in patients presenting to Swiss chiropractors compared to other countries. This is because chiropractic is one of the 5 government recognized medical professions in Switzerland. As a result, Swiss chiropractors are more likely to be referred patients earlier in the course of their symptoms compared to chiropractors in other countries [19].

### Limitations to the study

One of the limitations to this study is the short, two hour, test-retest time period used for the reliability part of this study. The excellent results obtained may be because the participants could remember their previous answers [17]. An attempt to inhibit this recall was done by changing the order of the domains on the BQ for the second administration of the questionnaire. This two hour time frame, however, is identical to the ones used in two previous translation and validation studies [13,16]. The fact that students with a mean age of approximately 22 years were used for the reliability part of the study is another limitation. It is unknown whether or not using a more heterogeneous age group would have influenced the reliability results. A further limitation is the fact that, like previous studies, this current study did not attempt to assess the content validity of this questionnaire [3,16,20]. This was done for the English version when it was originally created. Current methodology states that the content should also be evaluated when translating into a new language [7]. However, only chiropractic patients were used for the validation part of this study (another limitation). Comparison of chiropractic practice and patients in Switzerland, where this study occurred, with other countries was published in 2010 [19]. As the United Kingdom, where the BQ LBP questionnaire originated, was one of the countries with which Swiss chiropractors were compared, the content validity of this German version of the BQ should not be an issue. Because the validation of the BQ LBP questionnaire was only done using chiropractic patients as noted above, further testing with other practitioners and treatments should be done.

## Conclusions

The BQ for LBP was successfully translated and adapted into the German language. It was successfully tested for validity, consistency, and responsiveness against the German versions of the Oswestry Disability Index and the SF-36 questionnaire. The BQ LBP questionnaire is shorter than the other two questionnaires, covers more domains than the ODI and is more sensitive to change than the other questionnaires.

## Abbreviations

BQ: Bournemouth questionnaire; BT: Back translation; CI: Confidence interval; ICC: Intra-class correlation coefficient; LBP: Low back pain; ODI: Oswestry disability questionnaire; SD: Standard deviation; SRM: Standardized response mean.

## Competing interests

The authors declare that they have no competing interests.

## Authors’ contributions

CBF: Data acquisition, drafting manuscript, interpretation of data, approval of manuscript. CP: Concept and design of the study, analysis and interpretation of data, drafting and revising manuscript. JFM: Data acquisition, interpretation of data, revising and approval of manuscript. YL: Data acquisition, interpretation of data, revising and approval of manuscript. BKH: Concept and design of the study, revising manuscript, approval of manuscript. All authors read and approved the final manuscript.

## Supplementary Material

Additional file 1 Bournemouth Fragebogen für Patienten mit Rückenschmerzen. Click here for file
