# Supplementary material for: Translation and validation of the German version of the Bournemouth questionnaire for low back pain
Source: Chiropr Man Therap. 2013 Sep 26;21:32. doi: 10.1186/2045-709X-21-32 (PMC3849369; doi:10.1186/2045-709X-21-32)
Supplement: Additional file 1 — Bournemouth Fragebogen für Patienten mit Rückenschmerzen. [file 2045-709X-21-32-S1.pdf]

Bournemouth Fragebogen für Patienten mit Rückenschmerzen

Die folgenden Fragen wurden zusammengestellt, um mehr über Ihre Rückenschmerzen und deren Auswirkungen auf Sie herauszufinden. Bitte beantworten Sie ALLE Fragen, indem Sie auf der jeweiligen Skala dasjenige Kästchen unter der Zahl ankreuzen, (nur eine Zahl/Kästchen!) welches am besten beschreibt, wie Sie sich fühlen:

1. Wie stark würden Sie Ihre Rückenschmerzen der letzten Woche im Durchschnitt einstufen?

	0	1	2	3	4	5	6	7	8	9	10	
Gar keine Schmerzen	☐	☐	☐	☐	☐	☐	☐	☐	☐	☐	☐	Schlimmst mögliche Schmerzen

2. Wie stark wurde Ihr Tagesablauf letzte Woche von Ihren Rückenschmerzen beeinträchtigt? (Hausarbeit, Waschen, Ankleiden, Gehen, Treppensteigen, vom Bett beziehungsweise Stuhl aufstehen)

	0	1	2	3	4	5	6	7	8	9	10	
Gar nicht beeinträchtigt	☐	☐	☐	☐	☐	☐	☐	☐	☐	☐	☐	Konnte gar nichts erledigen

3. In welchem Ausmass haben Ihre Rückenschmerzen Sie während der vergangenen Woche daran gehindert, an Freizeit-/Familienaktivitäten oder anderen sozialen Aktivitäten teilzunehmen?

	0	1	2	3	4	5	6	7	8	9	10	
Teilnahme uneingeschränkt möglich	☐	☐	☐	☐	☐	☐	☐	☐	☐	☐	☐	Teilnahme nicht möglich

4. Wie unwohl/unruhig (verspannt; nervös; reizbar; Mühe, sich zu konzentrieren und zu entspannen) haben Sie sich während der vergangenen Woche gefühlt?

	0	1	2	3	4	5	6	7	8	9	10	
Gar nicht unwohl/unruhig	☐	☐	☐	☐	☐	☐	☐	☐	☐	☐	☐	Extrem unwohl/unruhig

5. Wie deprimiert (niedergeschlagen, traurig, pessimistisch, unglücklich) haben Sie sich während der vergangenen Woche gefühlt?

	0	1	2	3	4	5	6	7	8	9	10	
Überhaupt nicht deprimiert	☐	☐	☐	☐	☐	☐	☐	☐	☐	☐	☐	Extrem deprimiert

6. Wie stark hat Ihre Arbeit (zu Hause und am Arbeitsplatz) in der letzten Woche Ihre Rückenschmerzen beeinflusst?

	0	1	2	3	4	5	6	7	8	9	10	
Keine Verschlechterung der Rückenschmerzen	☐	☐	☐	☐	☐	☐	☐	☐	☐	☐	☐	Die Schmerzen wurden sehr viel schlimmer

7. In welchem Ausmass konnten Sie Ihre Rückenschmerzen in der vergangenen Woche selbstständig unter Kontrolle halten/vermindern?

	0	1	2	3	4	5	6	7	8	9	10	
Vollständige Kontrolle allein möglich	☐	☐	☐	☐	☐	☐	☐	☐	☐	☐	☐	Keine Kontrolle möglich

Datum:
